# Neurobehavioral abnormalities following prenatal psychosocial stress are differentially modulated by maternal environment

**DOI:** 10.1038/s41398-022-01785-5

**Published:** 2022-01-17

**Authors:** Sandra P. Zoubovsky, Michael T. Williams, Sarah Hoseus, Shivani Tumukuntala, Amy Riesenberg, Jay Schulkin, Charles V. Vorhees, Kenneth Campbell, Hee-Woong Lim, Louis J. Muglia

**Affiliations:** 1 Department of Pediatrics, University of Cincinnati College of Medicine, Cincinnati, OH USA; 2 Center for the Prevention of Preterm Birth, Perinatal Institute, Cincinnati Children’s Hospital Medical Center, Cincinnati, OH USA; 3 Molecular and Developmental Biology Graduate Program, University of Cincinnati College of Medicine, Cincinnati Children’s Hospital Medical Center, Cincinnati, OH USA; 4 Division of Neurology, Cincinnati Children’s Hospital Medical Center, Cincinnati, OH USA; 5 Division of Human Genetics, Cincinnati Children’s Hospital Medical Center, Cincinnati, OH USA; 6 Division of Developmental Biology, Cincinnati Children’s Hospital Medical Center, Cincinnati, OH USA; 7 Department of Neuroscience, Georgetown University, Washington, DC USA; 8 Department of Obstetrics and Gynecology, University of Washington, Seattle, WA USA; 9 Division of Neurosurgery, Cincinnati Children’s Hospital Medical Center, Cincinnati, OH USA; 10 Division of Biomedical Informatics, Cincinnati Children’s Hospital Medical Center, Cincinnati, OH USA; 11 Office of the President, Burroughs Wellcome Fund, Research Triangle Park, NC USA

**Keywords:** Molecular neuroscience, Psychiatric disorders

## Abstract

Prenatal stress (PS) is associated with increased vulnerability to affective disorders. Transplacental glucocorticoid passage and stress-induced maternal environment alterations are recognized as potential routes of transmission that can fundamentally alter neurodevelopment. However, molecular mechanisms underlying aberrant emotional outcomes or the individual contributions intrauterine stress versus maternal environment play in shaping these mechanisms remain unknown. Here, we report anxiogenic behaviors, anhedonia, and female hypothalamic-pituitary-adrenal axis hyperactivity as a consequence of psychosocial PS in mice. Evidence of fetal amygdala programming precedes these abnormalities. In adult offspring, we observe amygdalar transcriptional changes demonstrating sex-specific dysfunction in synaptic transmission and neurotransmitter systems. We find these abnormalities are primarily driven by in-utero stress exposure. Importantly, maternal care changes postnatally reverse anxiety-related behaviors and partially rescue gene alterations associated with neurotransmission. Our data demonstrate the influence maternal environment exerts in shaping offspring emotional development despite deleterious effects of intrauterine stress.

## Introduction

Substantial evidence from human and animal studies indicates that exposure to prenatal stress (PS) is a critical risk factor for developing neuropsychiatric disorders later in life [1–3]. However, the contributing mechanisms by which these intrauterine challenges program disease susceptibility remain largely unknown. Studies in rodents have demonstrated that PS can result in the emergence of anxiety-like and depressive-related behaviors, reduced social interaction, and deficits in attention and learning [4, 5]. These phenotypes are often accompanied by dysregulation in hypothalamic-pituitary-adrenal (HPA) axis activity [6], the main neuroendocrine system regulating responses to stress, and such dysregulation is a common feature in humans suffering from depression and anxiety disorders [7]. The effects of PS in rodents on offspring neurodevelopmental outcomes seem to be in part dependent on the type of stress experienced, timing of exposure, and offspring sex [1, 4, 8]. PS paradigms commonly utilized in animal studies rely on physical stressors, such as restraint, or do not accurately portray the multifaceted nature of stress experienced by women [9, 10]. As such, it becomes imperative to study the effects of gestational insults that are more translationally relevant, such as exposure to chronic variable psychosocial stressors.

Human and rodent studies have also demonstrated that PS is correlated with abnormalities in maternal behavior [11]. There is substantial evidence indicating that variations in maternal care can strongly influence offspring behavior and HPA axis activity [1, 12]. This raises the intriguing question of whether the neurodevelopmental programming effects of maternal stress are a consequence of in-utero disruptions or alterations in maternal care.

PS alters the developmental trajectory of vulnerable brain structures, resulting in functional changes that are thought to underlie the risk for developing emotional disorders [13]. The amygdala is a key site for integrating neuroendocrine and behavioral responses to stress and plays an essential role in emotion regulation [14]. Most research examining the effects of PS that are psychosocial in nature (exposure to social defeat) have focused on measuring gene expression changes in key molecular regulators of the HPA axis [4]. Upregulation in the glucocorticoid receptor (GR) and corticotropin-releasing hormone (CRH) have been noted, as well as altered CRH receptor (CRH R) levels [15–17]. While these findings underscore the importance of characterizing gene transcription changes in the amygdala to better understand the physiological basis of affective disturbances, sex-specific alterations in amygdalar transcriptional profiles in response to psychosocial PS and whether these changes arise in-utero or from alterations in maternal care remains to be investigated.

**Fig. 1:**
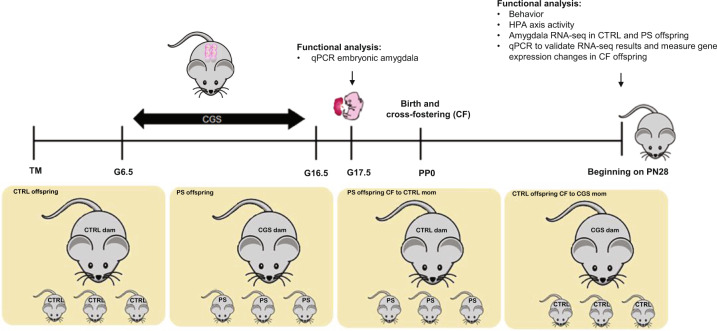
Schematic outlining of the experimental paradigm. Psychosocial stress was performed from gestational day 6.5 (G6.5) to G17.5. Fetal amygdala qPCR was performed on G17.5. A separate subset of mice was cross-fostered (CF) at birth so as to generate four separate groups for our studies. Functional analysis was carried out in these four groups beginning at postnatal (PN) day 28, including behavior and neuroendocrine characterization. RNA-seq analysis was performed in control and PS offspring amygdala samples and a subset of differentially expressed genes from this analysis were measured in cross-fostered offspring amygdala samples via qPCR.

To address these questions, we developed a chronic gestational stress (CGS) paradigm which consists of exposing pregnant mice to psychosocially challenging insults presented in an unpredictable fashion from gestational day 6.5–17.5. Employing these mild to moderate psychosocial manipulations allows us to better mimic stressors experienced by women during pregnancy [18]. We have previously found exposure to our paradigm results in the development of depression and anxiety-like phenotypes in dams as well as abnormalities in maternal behavior, evidenced by the emergence of fragmented and erratic maternal care patterns [19]. Here, we investigated the effects of psychosocial PS on offspring behavior and neuroendocrine function. To elucidate the molecular mechanisms underlying the various phenotypes observed, we compared amygdalar transcriptional profiles of control (CTRL) and PS offspring since the amygdala represents a nodal point between the HPA axis and emotional output. Lastly, to dissociate the impact of in-utero stress from abnormalities in maternal care resulting from stress during gestation, we performed cross-fostering (CF) of pups at birth between CTRL and CGS dams (Fig. 1).

## Materials and methods

### Animals

C57BL6/J mice were obtained from Jackson Laboratory. Mice were housed on a 14 h/10 h light–dark cycle with access to water and chow *ad libitum*. Female mice between 3 and 6 months of age were set up for timed-mating at 1800 h and separated the following morning at 0800 h. Simple randomization was used to divide mice with a copulatory plug, which was denoted as 0.5 days post-coitum, into two experimental groups (CTRL and CGS). All mouse experiments were in accordance with the guidelines of the National Institutes of Health and were approved by the Cincinnati Children’s Medical Center Animal Care and Use Committee.

### Psychosocial prenatal stress

Chronic psychosocial stress paradigm during pregnancy (CGS) was conducted as previously described [19]. Briefly, from gestational day (G) 6.5–16.5, mice assigned to the stress group were exposed to variable psychosocial insults 2 times per day, 2 h each, and an overnight stressor. Stressors included exposure to dirty rat bedding, foreign object (marble or lego) exposure, 30° cage tilt, bedding removal, frequent bedding changes, overnight lights on, overnight wet bedding, and overnight cage mate change. Control mice were not disturbed.

### Cross-fostering (CF)

Pups were switched with another litter within 24 h of birth, so as to generate four groups for our studies: CTRL offspring, PS offspring, PS offspring CF to CTRL mom, CTRL offspring CF to CGS mom.

### Offspring behavioral assessment

Behavioral tests were conducted in offspring when they reached postnatal day (PN) 28. One cohort underwent testing for anxiety-related behaviors using light–dark transition box (LD), followed by open field test (OFT) to assess for changes in locomotor activity, social interaction assay (SI) to measure alterations in sociability, and fear conditioning (FC) to quantify deficits in associative learning. A second cohort was used to assess behavioral coping strategy to stress using the forced swim test (FST). A third cohort was used to measure changes in anhedonia using the sucrose preference test (SPT). Experimenters were blinded to group membership.

*Light*–*dark transition box (LD)*. The LD was performed as previously described [20]. Mice were placed on the lighted side and the amount of time spent in each side of the apparatus, as well as number of crossings, was recorded over a 10 min period.

*Open field test (OFT).* The OFT was conducted as previously described [21]. Mice were tested for 1 h and locomotor activity was analyzed in 5 min intervals.

*Social interaction assay (SI).* SI was performed as previously described [22] with minor modifications. Amount of time spent interacting with the stranger mouse was used to quantify the degree of social interaction.

*Fear conditioning test (FC).* The FC assay which consisted of CS/US training and contextual and auditory cued components for fear conditioning was performed as previously described [23] in order to assess associative learning. Freezing behavior was quantified on day two (contextual fear testing) and three (cued fear testing).

*Forced swim test (FST).* The FST was performed as previously described [22]. Duration of immobility, as well as the frequency of immobility episodes, were recorded.

*Sucrose preference test (SPT).* The SPT was conducted as previously described [19]. Water and sucrose consumption (ml) were measured and preference was calculated using the average of the measurements from the last 4 days with the following formula: % preference = [(sucrose consumption/sucrose + water consumption) × 100].

### Serum corticosterone (CORT) measurements

In a separate cohort of mice, submandibular bleeds were performed at PN28 at circadian nadir, peak, and immediately following a 15 min swim in water (25 °C). Serum CORT measurements were performed by ELISA per manufacturer’s protocols (Arbor Assay, Ann Arbor, MI).

### Maternal behavior assessment

A separate cohort of mice was used for maternal behavior assessment, as previously described [19]. From postpartum day 2 to 5 (PP2-PP5), dams were observed for a 30 min period during the light cycle and the percentage of time spent nursing, licking/grooming pups, and off nest were recorded. The degree of fragmentation in maternal care was represented by the total number of licking/grooming bouts and the average length of an individual bout [19, 24]_._ The entropy rate was used to quantify the degree of unpredictability in maternal care, as previously described [19, 24–26]. Pup retrieval test was performed on PP6, as previously described [19]. Latency for dams to recover the first pup and the rest of the pups were recorded.

### Maternal milk corticosterone (CORT) measurements

Milk was collected from dams on PP9 as previously described [27]. Dams were separated from pups for 4 h prior to milking and anesthetized with ketamine (0.1 ml/20 g body weight ip) (Sigma, St. Louis, MO). Oxytocin (20 USP/ml ip) (Sigma) was injected to promote milk letdown and milk was collected 15 min after injection from each teat with capillary tubes (Drummond Scientific, Broomall, PA) and stored at −20 °C. Milk CORT measurements were performed by ELISA per the manufacturer’s protocol (Abcam, Cambridge, UK).

### Mouse tissue collection, amygdala microdissections, and RNA isolation

Pregnant female mice were euthanized on G17.5. Fetuses were collected. Tail tissue samples were used for genotyping to identify the sex of individual fetuses as previously described [16]. Brains from corresponding fetuses were collected and the ventrolateral portions of caudal telencephalic gross sections containing the amygdala were microdissected. For PN28 offspring, mice were sacrificed via cervical dislocations and brains were harvested, immediately frozen on dry ice, and stored at −80 °C. Amygdalar dissections were performed as previously described [28]. RNA from fetal brain and PN28 amygdalar dissections was purified using RNeasy Micro Kit (Qiagen, Hilden, Germany).

### quantitative PCR (qPCR)

RNA was converted to cDNA using the Quantitect Reverse Transcriptase Kit (Qiagen) and stored at −20 °C. qPCR was performed using Taqman system with Taqman Gene Expression Master Mix (ThermoFisher Scientific) on each cDNA sample as previously described [19] with minor modifications. Gene expression data were calculated using the ΔΔCt method.

### RNA-sequencing

RNA isolated from amygdalar microdissections from four control and four PS offspring (per sex) from different litters at PN28 was used for directional RNA-seq performed by the Genomics, Epigenomics, and Sequencing Core at the University of Cincinnati following previously published methods [29, 30]. The sequencing setting of single read 1 × 85 bp to generate ∼50 M reads per sample was used. Sequencing data have been deposited in NCBI’s Gene Expression Omnibus and are accessible through GEO Series accession number GSE189330.

### RNA-sequencing analysis

RNA-seq reads were aligned to mouse genome, mm10, using STAR aligner [31]. Raw reads counts aligned to each genes were measured using FeatureCounts [32]. Differentially expressed genes (DEGs) were analyzed using RUVseq [33] and EdgeR [34]. Genes with fold-change > 1.5 and FDR < 0.05 were selected as differential genes for gene ontology analysis using EnrichR [35].

### Statistics

Data were analyzed by mixed linear factorial ANOVA with degrees of freedom calculated using the Kenward-Roger method (Proc Mixed, SAS version 9.4, SAS Institute, Cary, NC, USA), two-way ANOVA test followed by Tukey’s post hoc test (Prism 7.0c software; GraphPad Software, Inc., San Diego, CA, USA), or unpaired two-tailed *t*-test (Prism 7.0c software) as indicated in figure legends. *P* ≤ 0.05 was considered significant. The n represents either offspring, litter numbers, or dams as indicated in figure legends. Results are reported as mean ± standard error of the mean (s.e.m.).

## Results

### Effects of psychosocial stress and cross-fostering on maternal behavior

We had previously published exposure to psychosocial stress during pregnancy results in abnormalities in the quality of maternal care, including fragmented and erratic maternal care patterns, as well as increased time retrieving pups in pup retrieval task [19], summarized in Table S1. To assess whether CF of offspring results in differences in maternal behavior, we examined the quantity and quality of maternal signals delivered by CTRL dams with CF PS offspring and CGS dams with CF CTRL offspring in the early postpartum period. No differences were observed in quantitative measures of maternal care, including percent time spent grooming CF offspring (Fig. S1A; t-test, *t*_16_ = 1.190, *P* = 0.2514), nursing CF offspring (Fig. S1B; t-test, *t*_16_ = 0.1082, *P* = 0.9152), or off-nest (Fig. S1C; t-test, *t*_16_ = 1.078, *P* = 0.2970), suggesting the overall duration of maternal care received was not affected by CF of offspring. However, CGS dams with CF CTRL offspring exhibited abnormalities in more qualitative aspects of maternal behavior, including increased fragmentation, defined by significantly shorter mean duration of licking/grooming bouts (Fig. S1D; t-test, *t*_16_ = 2.870, *P* = 0.0111) and a significant increase in the average number of bouts (Fig. S1E; t-test, *t*_16_ = 2.641, *P* = 0.0178), as well as a trend towards more unpredictable and chaotic maternal care patterns, as quantified by a trend towards an increased entropy rate (Fig. S1F; t-test, *t*_16_ = 1.798, *P* = 0.0910). We further assessed maternal care via the pup retrieval task. Although no differences were found in the latency to retrieve the first pup (Fig. S1G; t-test, *t*_16_ = 1.684, *P* = 0.1117), CGS dams with CF CTRL offspring displayed a significant increase in time needed to retrieve all pups (Fig. S1H; t-test, *t*_16_ = 2.526, *P* = 0.0225). These results are consistent with maternal behavior analysis previously performed [19] comparing control vs CGS dams without CF offspring, and indicate PS offspring CF to CTRL moms are experiencing changes in their postnatal maternal environment characterized by more consistent, less fragmented maternal signals when compared to CTRL offspring CF to CGS moms.

### Effects of psychosocial PS and changes in postnatal maternal environment on behavior

**Fig. 2:**
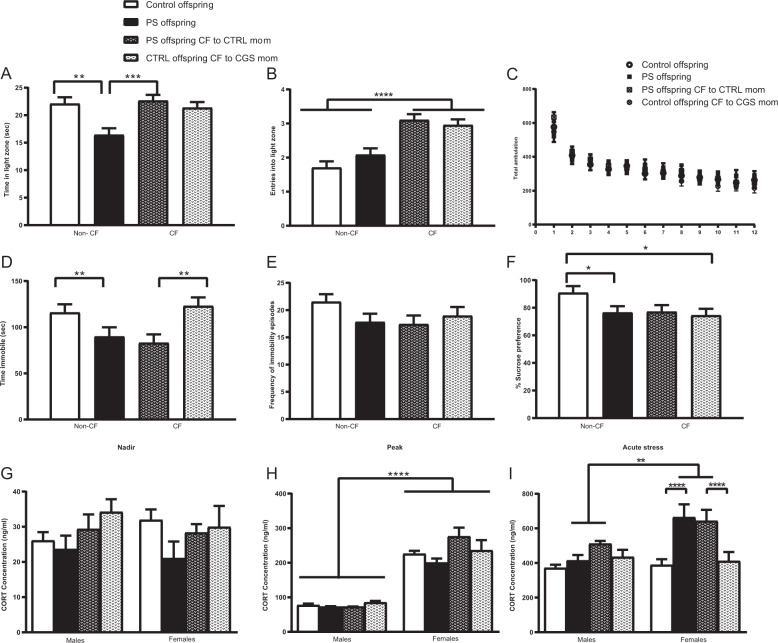
Psychosocial PS leads to behavioral abnormalities and female-specific neuroendocrine abnormalities that are differentially modulated by postnatal maternal environment. **A** Time spent and **B** entries into light compartment in LD, CTRL offspring = 30, PS offspring = 30, PS offspring CF to CTRL mom = 39, CTRL offspring CF to CGS mom = 40. **C** Total ambulation in OFT, CTRL offspring = 30, PS offspring = 30, PS offspring CF to CTRL mom = 39, CTRL offspring CF to CGS mom = 40. **D** Time spent immobile and **E** frequency of immobility episodes in FST, CTRL offspring = 38, PS offspring = 31, PS offspring CF to CTRL mom = 30, CTRL offspring CF to CGS mom = 29. **F** Percent sucrose preference as measured in the SPT, CTRL offspring = 28, PS offspring = 30, PS offspring CF to CTRL mom = 28, CTRL offspring CF to CGS mom = 28. Serum CORT measurements performed on PN28 at **G** nadir, **H** peak, and **I** after 15 min swim, CTRL offspring = 5–7, PS offspring = 5–7, PS offspring CF to CTRL mom = 6–7, CTRL offspring CF to CGS mom = 7, per sex per time point. Data presented as mean ± SEM. *p < 0.05, **p < 0.01, ***p < 0.001, ****p < 0.0001 mixed linear ANOVA with prenatal stress × cross-fostering × sex model and litter as a randomized block factor. For OFT, interval was a repeated measures factor.

We next measured the effects of psychosocial PS on anxiety-like behaviors via LD. A significant PS × CF interaction was detected in the total amount of time spent in the light zone of LD (Fig. 2A; *F*_1,27.7_ = 9.68, *p* = 0.0043). Offspring exposed to psychosocial PS showed a decrease in total time spent in the light zone when compared with age-matched CTRL offspring (*P* = 0.0023). This reduction was reversed in PS offspring CF to CTRL mothers (*P* = 0.0005), suggesting the emergence of an anxiety-like phenotype following PS that is significantly improved by changes in maternal care. No differences were observed between CTRL offspring and CTRL offspring CF to CGS mothers (*P* = 0.6456), indicating the fragmented maternal care patterns displayed by dams exposed to psychosocial stress during pregnancy [19] are not contributing to the emergence of anxiety-related behaviors in offspring. When evaluating the total number of entries into the light zone, a significant CF effect was detected (Fig. 2B; *F*_1,19.1_ = 40.44, *P* < 0.0001), where both PS offspring raised by CTRL mothers and CTRL offspring raised by CGS dams had more entries into the light zone. Despite these changes, analysis of locomotion in OFT revealed no significant effect of CF (Fig. 2C; *F*_1,26.9_ = 0.04, *P* = 0.8419) or PS effect (Fig. 2C; *F*_1,26.9_ = 0.48, *P* = 0.4940), suggesting that behavioral abnormalities or normalization of anxiety-like behaviors in PS offspring CF to CTRL mothers are not due to ambulatory changes.

In a separate experiment, we used the FST to assess for the type of coping strategy (active vs. passive) [36] displayed by PS offspring when being exposed to an acute inescapable stressor. We found a significant PS effect on the total amount of time spent immobile (Fig. 2D; *F*_1,30.6_ = 12.12, *P* = 0.0015). Interestingly, both PS offspring and PS offspring CF to CTRL mothers exhibited a significant reduction in immobility time when compared with respective CTRLs. We interpret this measure to reflect a more active coping strategy in PS offspring when faced with a stressor (e.g., swimming), which becomes significant as altered stress coping strategies have been found to be associated with a higher vulnerability for aberrant emotional outcomes, such as anxiety [37, 38]. Our data further suggest this alteration in stress coping strategy is a result of in-utero stress and not modulated by changes in postnatal environment (e.g., maternal care) as time spent immobile in CTRL offspring raised by CGS dams was similar to CTRL offspring raised by CTRL mothers (Fig. 2D; *F*_1,30.6_ = 0.54, *P* = 0.4673 for PS × CF interaction). No differences were observed across groups in the total number of immobility episodes (Fig. 2E; *F*_1,25_ = 2.91, *P* = 0.1006 for PS effect; *F*_1,25_ = 0.91, *P* = 0.3487 for CF effect). We used the SPT to evaluate anhedonia, a symptom often seen in depression [39]. A significant PS × CF interaction was observed on the preference for 4% sucrose solution (Fig. 2F; *F*_1,26.9_ = 3.24, *p* = 0.0416). PS offspring displayed significantly reduced preference for 4% sucrose than age-matched CTRL offspring (*P* = 0.0396), a measure considered to represent an inability to experience pleasure. Interestingly, the emergence of this anhedonic behavior seemed to be mediated by both effects of in-utero stress and alterations in postnatal maternal environment as CTRL offspring raised by stressed mothers also exhibited a reduction in sucrose preference when compared with non-CF CTRLs (*P* = 0.0221). Our data suggest that anhedonic characteristics could not be rescued by normalizing maternal care delivered to pups as PS offspring CF to CTRL dams exhibited a similar reduction in sucrose preference as non-CF PS offspring (*P* = 0.9252). Noteworthy, no significant sex × PS interaction was found on the amount of time spent in the light zone in LD (Fig. 2A; *F*_1,105_ = 0.00, *P* = 0.9951), on the amount of time spent immobile in the FST (Fig. 2D; *F*_1,93.9_ = 1.62, *P* = 0.2064), and on 4% sucrose preference in the SPT (Fig. 2F; F_1, 84.9_ = 0.00, *P* = 0.9816), indicating male and female PS offspring were equally affected in the behavioral parameters measured by these assays.

There were no differences in the total amount of time spent socializing with a stranger mouse in SI in PS offspring when compared with CTRLs (Fig. S2A) or in associative learning as measured by no changes in freezing behavior during FC (Fig. S2B−D).

### Effects of psychosocial PS and changes in postnatal maternal environment on neuroendocrine function

In order to determine the effects of psychosocial PS on neuroendocrine function, we measured circadian concentrations in serum CORT levels and in response to a novel acute stressor at PN28. No differences were observed in CORT measurements at the circadian nadir timepoint (Fig. 2G; *F*_1,21_ = 2.92, *P* = 0.1023 for PS effect; *F*_1,21_ = 0.03, *P* = 0.8598 for sex effect; *F*_1,21_ = 0.22, *P* = 0.6462 for PS × sex interaction). Analysis of peak CORT values revealed no effect of PS (Fig. 2H; *F*_1,20.3_ = 0.00, *P* = 0.9631), although there was a significant main effect of sex (Fig. 2H; *F*_1,22_ = 198.13, *p* < 0.0001). Increased CORT was measured at the peak timepoint in females across all our groups when compared with males, a normal sex-variation often noted in HPA axis activity [40]. Following 15 min of swimming, there was a significant PS × sex interaction on serum CORT levels (Fig. 2I; *F*_1,105_ = 6.39, *P* = 0.0129). Elevation in stress-induced CORT secretion was observed in PS females when compared with PS males (*P* = 0.0012). Importantly, PS females and PS females CF to CTRL dams had similar serum CORT levels in response to the 15 min swim, which were significantly higher than CTRL females and CF CTRL females (*P* < 0.0001).

**Fig. 3:**
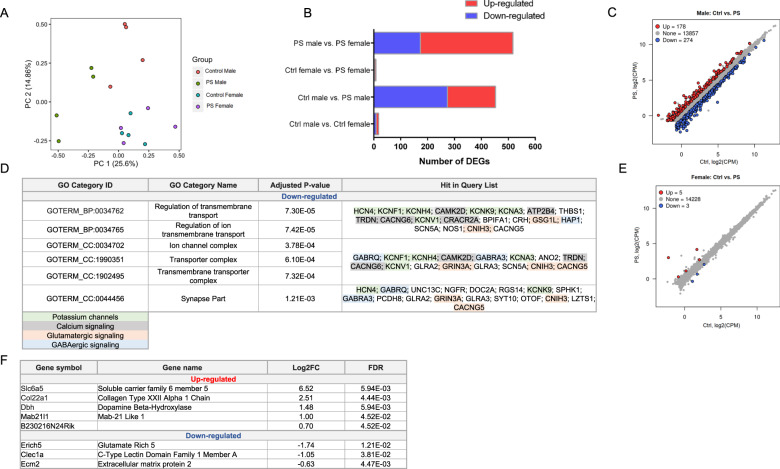
Psychosocial PS results in sexually dimorphic gene expression changes in offspring amygdala. **A** Principal component analysis plot. **B** Total number of differentially expressed genes for the various pair-wise comparisons made, CTRL offspring = 4 per sex, PS offspring = 4 per sex. **C** Scatter plot displaying 178 upregulated and 274 downregulated genes in PS males when compared with CTRL males. Genes were considered significant with an FDR < 0.05 and FC > 1.5. **D** Significantly enriched pathways after gene ontology analysis of downregulated genes using biological process and cellular component categories. Genes significantly downregulated in the amygdala of PS males when compared to CTRL males include genes associated with potassium channels, calcium signaling, glutamatergic signaling, and GABAergic signaling. **E** Scatter plot displaying 5 upregulated and 3 downregulated genes in PS females when compared with CTRL females. **F** Summary of DEGs. Genes were considered significant with an FDR < 0.05 and FC > 1.5.

### Effects of cross-fostering on maternal milk composition

To address if changes in maternal milk composition could be partially underlying programming of offspring brain development and behavior, we measured milk CORT concentration at PP9 between CGS and CTRL dams. No significant differences in CORT milk were observed between these two groups (Fig. S3; t-test, *t*_16_ = 1.400, *P* = 0.1805), suggesting changes in quality of maternal care as opposed to milk CORT content are contributing to the offspring behavioral changes being observed after CF.

### Effects of psychosocial PS on fetal amygdala

To examine the possible effects of psychosocial PS on fetal brain development, we investigated whether changes in gene expression could be measured in the amygdala, a key brain region for emotional output, at E17.5. We focused on molecular regulators of the HPA axis as previous studies have shown these to be altered by PS [4]. We observed a trend towards a significant main effect of PS on CRH mRNA levels (Fig. S4A; *F*_1,4_ = 5.39, *P* = 0.0810). CRH levels were elevated in PS fetuses when compared with CTRLs, although this difference was not statistically significant. We also detected a trending effect of PS on amygdalar GR expression (Fig. S4B; *F*_1,4_ = 6.84, *P* = 0.0591). PS fetuses exhibited reduced but not statistically significant GR mRNA levels when compared with CTRL fetuses. There was a significant main effect of PS on CRH R1 expression (Fig. S4C; *F*_1,4_ = 16.14, *P* = 0.0159). Psychosocial PS exposure resulted in decreased amygdalar CRH R1 mRNA levels in E17.5 fetuses. Analysis of CRH R2 expression revealed no significant effect of PS (Fig. S4D).

### Effect of psychosocial PS on amygdalar transcriptomes

To investigate the molecular mechanisms underlying sex-specific effects of psychosocial PS on behavior and neuroendocrine function, we performed RNA-seq on PN28 amygdalar samples from CTRL and PS male and female offspring. Principal component analysis revealed that samples from PS mice were distinguishable from CTRL mice more significantly in male than in female samples (Fig. 3A). To examine detailed transcriptomic changes, we performed DEG analysis using four pairwise comparisons: CTRL male vs. PS male, CTRL female vs. PS female, CTRL male vs. CTRL female, and PS male vs. PS female. DEGs were determined by FDR < 0.05 and FC > 1.5. Indeed, male mice displayed much larger number of DEGs after PS exposure than females (Fig. 3B).

In the CTRL male vs. CTRL female amygdala comparison, there were 18 DEGs, with 9 female-specific and 9 male-specific genes (Fig. S5A). Transcripts displaying the largest effect were localized on sex chromosomes (e.g., *Xist, Eif2s3y*) (Fig. S5B), and previously reported to be differentially expressed between the sexes in the hypothalamus [41]. Gene ontology (GO) analysis for the DEGs in this comparison revealed enrichment in genes encoding for calcium-binding proteins associated with a wide variety of processes, including inflammation (RAGE receptor binding) and energy metabolism (long chain fatty acid binding) (Fig. S5C).

In the CTRL male vs. PS male comparison, we identified a total of 452 DEGs with 178 upregulated and 274 downregulated (Fig. 3C). According to GO analysis, we observed that genes related to the regulation of transmembrane transport and components of the synaptic membrane were significantly downregulated in PS males. The majority were genes associated with potassium channels (including *KCNF1, KCNH4, KCNK9, KCNA3,* and *KCNV1*), genes associated with calcium signaling (*ATP2B4, TRDN,* and *CACNG6*, *CRACR2A,* and *CAMK2D*), genes implicated in glutamatergic signaling (*GSG1L, CNIH3, CACNG5,* and *GRIN3A*), and GABAergic signaling (*GABRQ, GABRA3,* and *HAP1*) (Fig. 3D). Although GO analysis among the upregulated genes in PS males did not reveal any significant pathway enrichment (data not shown), several of the significantly upregulated genes were also associated with glutamatergic signaling (including *GRM2, GRM4, HOMER 3, TCF7L2,* and *GRID2IP*).

Interestingly, only a small subset of genes were differentially expressed in PS females compared with CTRL females. This comparison yielded only a total of 8 DEGs, with 5 upregulated and 3 downregulated (Fig. 3E). Among the genes displaying the largest effects were *SLC6A5*, involved in glycine neurotransmitter uptake, and *DBH*, which encodes the rate-limiting enzyme for norepinephrine (NE) biosynthesis [42], which were significantly upregulated after PS (Fig. 3F).

In the final comparison, PS male vs. PS female, we identified 517 DEGs, with 344 PS female-specific and 177 PS male-specific genes (Fig. S5D). GO analysis with the biological process and cellular component categories confirmed alterations in synaptic transmission, transmembrane transport, and glutamatergic receptor signaling, as noted above, with genes under these categories significantly expressed in PS females when compared with PS males (Fig. S5E).

### Effects of changes in postnatal maternal environment on amygdalar gene expression

**Fig. 4:**
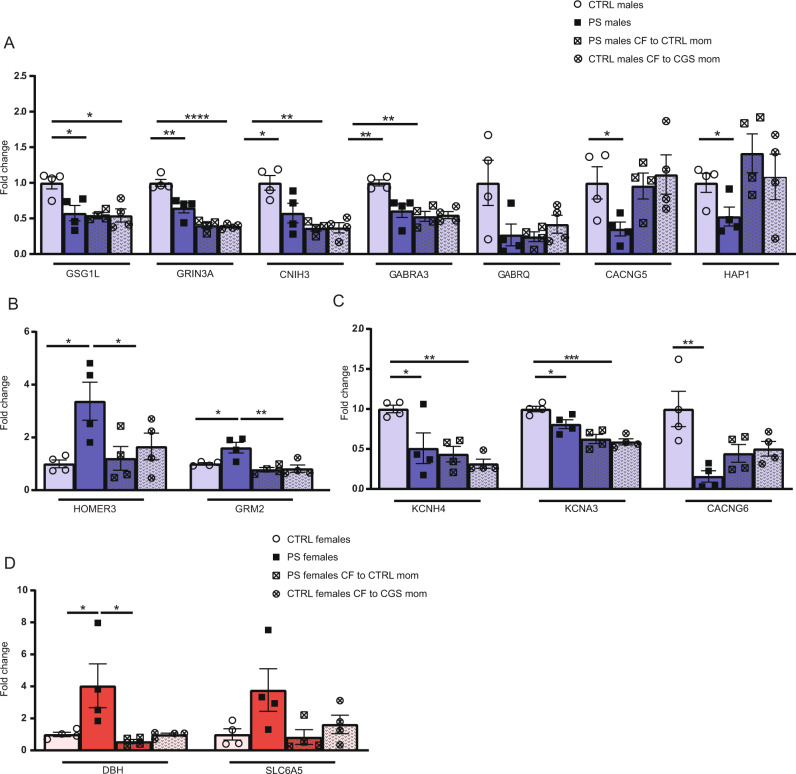
Validation of RNA-seq results through qPCR and effects of CF on gene expression. **A** Validation of RNA-seq results for a subset of genes confirms downregulation in genes associated with glutamatergic and GABAergic neurotransmitter systems in PS males that are not reversed by changes in postnatal maternal environment. **B** Reversal of genes associated with metabotropic glutamate neurotransmission are observed in PS males following CF. **C** qPCR data also confirms downregulation of genes encoding ion transporter complexes in PS males which are not reversed by alterations in maternal environment. **D** Validation of RNA-seq results in PS females confirms upregulation in DBH and a trend towards increased levels in SLC6A5 transporter. CF induces normalization of alterations in DBH in PS females, associated with NE neurotransmission. *N* = 4 per group. Data presented as mean ± SEM. *p < .05, **p < 0.01, ***<*p* < .001, ****p < 0.0001, unpaired two-tailed *t*-test for differences between Ctrl and PS offspring, *t*wo-way ANOVA followed by Tukey’s post hoc test for CF gene expression analysis with PS × CF model.

Several of the genes associated with synaptic transmission were chosen for qPCR validation. In PS males, we were able to confirm significant alterations of genes encoding proteins involved in glutamatergic (downregulation of *GSG1L, GRIN3A, CNIH3,* and *CACNG5*, and upregulation *of HOMER 3 and GRM2)* and GABAergic neurotransmission (downregulation of *GABRA3A*) (Fig. 4A, B). Downregulation of genes associated with ion channel complexes was also confirmed (*KNCH4, KCNA3,* and *CACNG6*) (Fig. 4C). *GABRQ* was the only gene chosen for validation via qPCR which exhibited a trend towards downregulation after PS in males (*P* = 0.0837), a direction of change consistent with RNA-sequencing results, but that did not reach statistical significance (Fig. 4A). In females, there was a significant upregulation in DBH after PS, and a trend towards increased levels of *SLC6A5* (Fig. 4D; *P* = 0.0899).

To differentiate the contribution of in-utero stress from maternal environment on gene transcription, we measured how CF altered PS-induced gene expression changes. We observed that most of the genes downregulated in PS males were also downregulated in CTRL males CF to CGS moms, suggesting both in-utero stress and alterations in maternal care are contributing to transcriptional changes noted in our study (Fig. 4A, C). Importantly, in PS males CF to CTRL moms, we observed a partial rescue of alterations in genes associated with glutamatergic signaling. We find expression levels of *HOMER3* (Fig. 4B; *F*(1,12) = 7.990; *P* = 0.0153 PS × CF interaction; post hoc *P* < 0.05) and *GRM2* (Fig. 4B; *F*(1,12) = 6.178; *P* = 0.0287 PS × CF interaction; post hoc *P* < 0.01) are normalized following CF, and note a trend towards normalization of *CACNG5* and *HAP1* (Fig. 4A), suggesting a partial rescue in metabotropic glutamatergic neurotransmission could underlie the reversal of anxiety-related behaviors in PS males CF to CTRL moms. In PS females, we observe expression of *DBH* is normalized following CF to CTRL moms (Fig. 4D; *F*(1,12) = 6.235; *P* = 0.0281 PS × CF interaction; post hoc *P* < 0.05), suggesting a partial rescue in NE neurotransmission could underlie the reversal of anxiogenic behaviors in PS females CF to CTRL moms.

Our results are summarized in Table S2.

## Discussion

In our study, we find psychosocial PS results in the emergence of anxiety-like behaviors, an altered behavioral coping strategy to stress, and anhedonia in male and female offspring. Neuroendocrine abnormalities, evidenced by acute stress-induced HPA axis hyperactivity, are only observed in PS female offspring. We further demonstrate these abnormalities are differentially modulated by changes in maternal care postnatally, with only anxiety-related behaviors rescuable by CF to CTRL mothers. In addition, we find evidence that these abnormalities are preceded by gene expression changes in the fetal amygdala, suggesting programming of brain development leading to increased susceptibility to emotional disturbances. The molecular analysis further revealed phenotypic changes in PS offspring to be associated with sex-specific disturbances in genes associated with synaptic transmission, including genes associated with glutamatergic and GABAergic signaling in PS males, and upregulation of *DBH*, known to regulate NE synthesis [42], in PS females. Lastly, CF of PS offspring to CTRL mothers partially normalizes changes in gene expression related to glutamatergic and NE signaling, which may contribute to the reversal of anxiety-like behaviors.

Our data indicate exposure to psychosocial PS leads to anxiety-like behaviors and anhedonia in male and female offspring. In contrast, only PS female offspring exhibit HPA axis dysregulation. Consistent with our data, an abundance of studies have shown PS exposure results in depressive behaviors and anhedonia in offspring of both sexes [10]. However, previous studies reporting the effects of PS on anxiety-related behaviors and neuroendocrine dysfunction are not always consistent. PS paradigms with a psychosocial component have demonstrated anxiety-like behaviors and HPA axis hyperactivity in male offspring [15, 16, 43], with males being particularly susceptible to effects of PS experienced during early gestation [16]. Interestingly, increased anxiety and stress-induced HPA axis hyperactivation are reported in PS female offspring if stress paradigms with a psychosocial component are applied during middle to late gestation [44, 45]. These results suggest sex-specific windows of vulnerability to psychosocial PS, with females becoming susceptible to HPA axis dysfunction and the development of anxiety during mid-to-later stages of fetal development. These sex-dependent effects of PS could partially be explained by the developmental timing of relevant processes, including a greater increase in limbic GR expression in female brains during later in-utero life [46].

Our findings further demonstrate both PS male and female fetuses exhibited changes in amygdalar gene expression. Psychosocial PS was associated with decreased CRHR1 mRNA levels in both sexes and trends towards upregulation of CRH and downregulation of GR expression. These results suggest the protective glucocorticoid barrier in the placenta [47] is being overcome by excess glucocorticoids resulting in fetal overexposure to CORT and alterations in brain developmental trajectories. An additional contributor might include catecholamines, stress-related amine hormones known to regulate various placental processes and influence brain development [47].

**Fig. 5:**
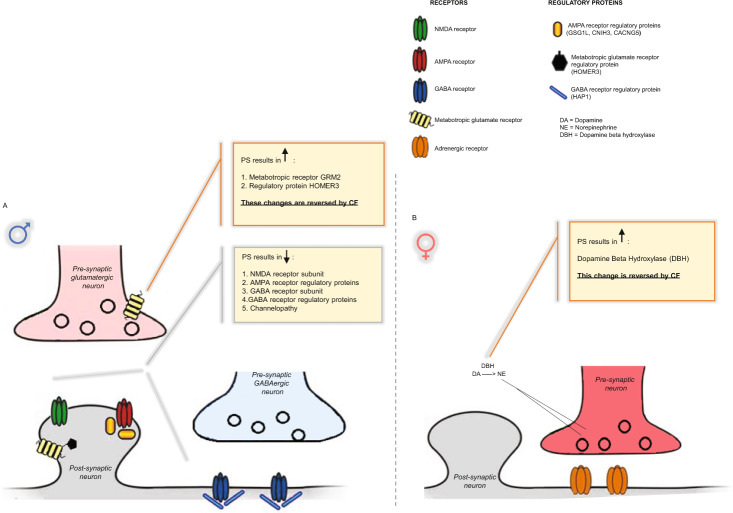
Model summarizing gene expression changes in PS offspring and normalizations induced by CF. **A** In males, psychosocial PS results in downregulation genes encoding proteins associated with glutamatergic signaling, GABAergic signaling, and ion channel transporter complexes. These transcriptional changes associated with synaptic transmission are likely resulting in an imbalance in excitatory and inhibitory transmission, resulting in the behavioral abnormalities observed in PS males. CF to CTRL mothers normalizes gene expression changes associated with metabotropic glutamate receptor signaling and reverses anxiety-related behaviors. **B** In females, PS results in an upregulation of DBH, which is likely to increase noradrenergic tone and partially underlie HPA axis hyperactivity and increased anxiety noted in PS females. CF normalizes DBH expression and reverses anxiety-related behaviors in PS females.

The amygdala undergoes dramatic structural and functional modifications as a result of stress [48, 49]. Here, we examined amygdalar transcriptional responses following psychosocial PS. PS males exhibited alterations in genes involved in synaptic transmission, including downregulation of voltage-gated potassium channels and calcium signaling genes. In addition, we detected alterations in transcripts encoding synaptic proteins involved in glutamatergic and GABAergic neurotransmission. Our findings are consistent with other studies examining the effects of prenatal insults on amygdalar gene expression, which have observed changes in ion transporter complexes, GABA_A_ receptor subunits, as well as ionotropic and metabotropic glutamate receptors [50–52]. Our data, however, identify a number of novel PS responsive transcripts, including genes encoding regulatory proteins known to modulate AMPA receptor trafficking and channel kinetics, such as *GSG1L*, *CACNG5*, and *CNIH3* [53, 54], and genes encoding proteins involved in postsynaptic stabilization of metabotropic glutamate receptors, such as *HOMER3*, and GABA_A_ receptor trafficking partner *HAP1* (Fig. 5) [55, 56]. Proper excitatory and inhibitory neurotransmission balance is crucial for preventing amygdala hyperactivity, a neurological feature often noted in patients suffering from emotional disorders [57–60]. The complex pattern of gene changes associated with synaptic transmission detected in our study could be affecting the balance of excitatory and inhibitory transmission within the amygdala and resulting in the behavioral abnormalities observed in PS males. Unexpectedly, only minor changes were observed in PS females, including increased expression of dopamine beta hydroxylase (*DBH*), a gene associated with NE synthesis [42] (Fig. 5). Stress induced increases in *DBH* can result in increased noradrenergic tone and partially underlie the enhanced stress sensitivity and increased anxiety noted in PS females [61, 62]. Additionally, other brain regions known to exhibit a sexually dimorphic response to stress, such as the bed nucleus stria terminalis [63, 64], could be more robustly affected.

A primary objective of our study was to dissociate in-utero from postnatal maternal environmental effects. In line with other studies, our data indicate that in-utero stress contributes to the emergence of anxiety-related behaviors, anhedonia, and HPA axis dysfunction [4, 65, 66]. Interestingly, we find these abnormalities are differentially affected by changes in maternal environment. Anxiety-like behavior exhibited by PS offspring is rescued by CF to CTRL mothers who exhibit less fragmented maternal care patterns than CGS dams, suggesting neural circuit alterations underlying this phenotype are ameliorated by changes in postnatal maternal care. Given no differences were observed in maternal milk CORT concentrations in the early postpartum period between CTRL and CGS dams with CF offspring, these results suggest changes in behavioral phenotype are likely due to changes in quality of maternal care received, as opposed to CORT content in maternal milk. Nevertheless, further studies are needed to determine if differences in nutritional content or other milk bioactive molecules [27] could be implicated in the reversal of programming of anxiety-like behaviors.

We further observed normalization of amygdalar *HOMER3* and metabotropic glutamate receptor *GRM2* expression following CF of PS males with CTRL dams, as well as the rescue of *DBH* expression in PS females CF to CTRL moms (Fig. 5). These results suggest CF-induced changes in metabotropic glutamate signaling and NE signaling could be partially restoring the balance in excitatory and inhibitory transmission within the amygdala of PS offspring, thus ameliorating anxiogenic behaviors. Previous studies manipulating the quality of maternal care by neonatal handling have shown the reversal of both anxiogenic behavior and HPA axis hyperactivity following PS [5, 67, 68]. In our study, cross-fostering was not effective in rescuing the enhanced HPA axis drive and altered stress coping strategy noted in PS offspring, suggesting the influence of psychosocial PS on these phenotypes differs from the effect of other types of prenatal insults, with psychosocial PS being associated with more persistent deleterious alterations. Lastly, the emergence of anhedonia in PS offspring observed in our study seemed to be mediated both by in-utero stress and abnormalities in maternal care, and was not ameliorated with CF. Gene expression alterations not normalized by CF might be contributing to these phenotypes, including downregulation of several regulatory proteins associated with AMPA receptor trafficking and ion channel complexes in PS males.

In conclusion, we find psychosocial PS results in behavioral and sex-specific neuroendocrine abnormalities that are differentially modulated by changes in maternal care. These abnormalities are associated with a complex pattern of gene expression changes in the amygdala indicating dysfunctions in neurotransmitter systems, with pronounced sex differences. In addition, we find CF triggers changes in genes associated with glutamatergic and NE neurotransmission that are associated with the reversal of anxiety-related behaviors. Studies aimed at interrogating the role specific CF altered genes play in rescuing anxiogenic behaviors could provide opportunities for the development of novel, clinically relevant therapeutic strategies.

## Supplementary information

Supplementary Information Text

Supplementary table 1

Supplementary figure 1

Supplementary figure 2

Supplementary figure 3

Supplementary figure 4

Supplementary figure 5

Supplementary table 2
