# Supplementary material for: Neurobehavioral abnormalities following prenatal psychosocial stress are differentially modulated by maternal environment
Source: Transl Psychiatry. 2022 Jan 17;12:22. doi: 10.1038/s41398-022-01785-5 (PMC8764031; doi:10.1038/s41398-022-01785-5)
Supplement: Supplementary file 1 — Supplementary Information Text [file 41398_2022_1785_MOESM1_ESM.docx]

**Supplementary Information:**

**Supplementary material and methods:** Complete description

**Supplementary figure:**

Supplementary Table 1: Summary of maternal behavior analysis previously assessed in CTRL vs CGS dams.

Supplementary Figure 1: Maternal behavior analysis in dams with CF offspring.

Supplementary Figure 2: Psychosocial PS has no effect on offspring sociability or associative learning.

Supplementary Figure 3: Maternal milk CORT concentrations in dams with CF offspring.

Supplementary Figure 4: Effects of psychosocial PS on fetal brain development.

Supplementary Figure 5: Analysis of DEGs in the amygdala in CTRL females vs. CTRL males and PS males vs. PS females.

Supplementary Table 2: Comparison of fold change determined by RNA-seq and qPCR for a subset of genes selected for validation of RNA-seq and summary of CF-induced changes.

**Supplementary Material and Methods**

**Animals** C57BL6/J mice were obtained from Jackson Laboratory. Mice were housed on a 14-hour/10-hour light-dark cycle (lights on at 6:00 A.M.) with access to water and chow *ad libitum*. After being allowed to habituate to the animal facility for at least 2 weeks, virgin female mice between 3 and 6 months of age were set up for timed-mating at 1800h and separated the following morning at 0800h. Simple randomization was used to divide female mice with a copulatory plug, which was denoted as 0.5 days post-coitum, into two experimental groups (CTRL and stressed (CGS)), and housed in groups of four. All mouse experiments were in accordance with the guidelines of the National Institutes of Health and were approved by the Cincinnati Children’s Medical Center Animal Care and Use Committee.

**Psychosocial prenatal stress** Chronic psychosocial stress paradigm during pregnancy (CGS) was conducted as previously described^1^. Briefly, from gestational day (G) 6.5 to 16.5, mice assigned to the stress group were exposed to variable psychosocial insults 2 times per day, 2 h each, and an overnight stressor. Control mice were not disturbed.

**Cross-fostering (CF)** Pups were switched with another litter within 24h of birth, so as to generate four groups for our studies: CTRL offspring, PS offspring, PS offspring CF to CTRL mom, CTRL offspring CF to CGS mom.

**Offspring Behavioral Assessment** Behavioral tests were conducted from least to most stressful in offspring when they reached postnatal day (PN) 28. Three separate cohorts were used for behavioral testing. One cohort underwent testing for anxiety-related behaviors using light-dark transition box (LD), followed by open field test (OFT) to assess for changes in locomotor activity, social interaction assay (SI) to measure alterations in sociability, and fear conditioning (FC) to quantify deficits in associative learning at the Cincinnati Children’s Research Foundation’s Animal Behavior Core. A second cohort was used to assess behavioral coping strategy to stress using the forced swim test (FST). A third cohort was used to measure changes in anhedonia using the sucrose preference test (SPT). To control for litter effects, one to two males or one to two females per litter selected by simple randomization were tested. Statistically, litter was a randomized block factor during analysis. Experimenters were blinded to group membership.

*Light-dark transition box (LD)* The LD was performed as previously described^2^. Briefly, offspring were placed inside the apparatus used for OFT which was modified with a black acrylic insert

that divided the chamber into two sides, one dark and one light, each measuring (20.5 cm x 41 cm) with a 7 x 7 cm opening between them. Mice were placed on the lighted side and the amount of time spent in each side of the apparatus as well as number of crossings was recorded over a 10-min period.

*Open field test (OFT)* The OFT was conducted as previously described^3^. Briefly, mice were placed inside an activity chamber measuring (41 cm x 41 cm x 38 cm) (San Diego Instruments, San Diego) with 16 photobeams spaced 2.5 cm apart in the x and y planes. Mice were tested for 1h and locomotor activity was analyzed in 5-min intervals.

*Social interaction assay (SI)* SI was performed as previously described^4^ with minor modifications. A three-chamber clear acrylic apparatus divided in three compartments was used. Two larger compartments (30 x 70 cm each) and a smaller central compartment (8.5 x 70 cm) were connected by openings in the center of each partition (7.5 x 5 cm). The larger compartments contained a small circular confinement cage each (measuring 15 cm in diameter). The test consisted of three parts conducted on a single day. Mice were first placed in the center compartment without access to the larger chambers and allowed to explore for 5 min. Next, mice were allowed to explore all three chambers of the apparatus for 5 min. Finally, in the testing phase, a stranger mouse was introduced (an unfamiliar conspecific of the same strain and sex) into one of the two confinement cages and the other confinement cage remained empty. Mice were allowed to explore freely for 5 min and movement was tracked using ANYmaze software (Stoelting, Wood Dale, IL). Amount of time spent interacting with the stranger mouse was used to quantify the degree of social interaction.

*Fear conditioning test (FC)* The FC assay which consisted of CS/US training and contextual and auditory cued components for fear conditioning was performed as previously described^5^ in order to assess associative learning. The apparatus contained a grid floor connected to a scrambled foot shock device mounted inside a sound-attenuated chamber (San Diego Instruments, San Diego, CA). On day one, mice were placed inside the apparatus for the training phase where they received three tone-footshock pairings spaced 180 s apart (tone: 82 dB, 2 kHz, 30 s duration; shock 1 s, 0.3 mA) near the end of a 10 min period. On day two, for contextual fear testing, mice were placed back in the chamber for 6 min without auditory cues. On day three, for cued fear testing, the grid floor inside the chamber was replaced with a different floor with a black hexagonal insert and mice were placed in the chamber for 3 min with no tone present followed by 3 min of tone. Freezing behavior was quantified on day two (contextual fear testing) and three (cued fear testing).

*Forced swim test (FST)* The FST was performed as previously described^4^. Briefly, mice were placed in a 2 L beaker with 1.5 L of water acclimatized to room temperature (25°C). On day 1, the habituation session, mice were placed in the beaker for 15-min. The testing session occurred the following day. Mice were allowed to swim for 5-min and duration of immobility as well as frequency of immobility episodes were recorded. Immobility was defined as lack of all motion except the minimal movement required to keep the mouse afloat.

*Sucrose preference test (SPT)* The SPT was conducted as previously described^1^. Briefly, mice were single housed with *ad libitum* chow, and given free access to one 100 ml graduated bottle containing tap water and another 100 ml bottle containing 4% sucrose for 6 days. The position of the bottles was interchanged daily to reduce side-bias. Water and sucrose consumption (ml) were measured daily and preference was calculated using the average of the measurements from the last 4 days only with the following formula: % preference = [(sucrose consumption/sucrose + water consumption) × 100].

**Serum corticosterone (CORT) measurements** In a separate cohort of mice, submandibular bleeds were performed at PN28 at circadian nadir, peak, and immediately following a 15-min swim in water acclimatized to room temperature (25°C). The blood was collected in serum separator tubes, centrifuged at 21,130 x g for 6-min, and serum was removed and stored at -20⁰C. Serum CORT measurements were performed by ELISA per manufacturer’s protocols (Arbor Assay, Ann Arbor, MI).

**Maternal behavior assessment** A separate cohort of mice was used for maternal behavior assessment, as previously described^1^. Pregnant mice were exposed to CGS or controls left undisturbed. Within 24h of birth, pups were CF so as to generate two groups, CTRL mom with PS offspring and CGS mom with CTRL offspring, and litters were culled to 6 pups. From postpartum day 2 to 5 (PP2-PP5), CTRL and CGS dams with CF offspring were observed for a 30-min period during the light cycle and percentage of time spent nursing, licking/grooming pups, and off nest were recorded. Degree of fragmentation in maternal care was represented by the total number of licking/grooming bouts and the average length of an individual bout^1,6^_._ Entropy rate was used to quantify degree of unpredictability in maternal care, as previously described^1,6-8^. Dams were categorized as performing different types of behavior: licking/grooming pups, nursing, nest building, self-grooming, carrying pups, or off-nest. The proportion of times a dam was observed transitioning from one behavior to the next was calculated for each dam. Following previously published protocols^1,6-8^, a first-order Markov chain was subsequently used to model the behavioral sequences and to calculate the entropy rate, or degree of randomness in maternal behavior sequences, using the formula$E$ $=-\sum_{i=1}^{M} \pi_{i}\sum_{j=1}^{M} p_{ij}log\left( p_{ij} \right)$, where p_ij_ measures the proportion of times the dam was recorded as transitioning from behavior *i* to behavior *j* and M=6 is the total possible number of behaviors, and $\pi_{i}$ was defined as the proportion of time a dam was exhibiting each behavior. Pup retrieval test was performed on PP6, as previously described^1^. Dams were removed from their home cage. Pups were randomly distributed across the cage and latency for dams to recover the first pup and and the rest of the pups to the original nest position were recorded.

**Maternal milk corticosterone (CORT) measurements** Milk was collected from dams on PP9 as previously described^9^. Dams were separated from pups for 4h prior to milking, anesthesized with ketamine (0.1 ml/20 g body weight ip) (Sigma, St. Louis, MO). Oxytocin (20 USP/ml ip) (Sigma) was injected to promote milk letdown and milk was collected 15 minutes after injection from each teat with capillary tubes (Drummond Scientific, Broomall, PA) and stored in microcentrifuge tubes at -20*C. Milk CORT measurements were performed by ELISA per manufacturer’s protocol (Abcam, Cambridge, UK).

**Mouse tissue collection, amygdala microdissections, and RNA isolation** Pregnant female mice were euthanized on G17.5. Following laparotomy, the fetuses were collected. Tail tissue samples were used for genotyping to identify sex of individual fetuses with primers specific for SRY (5’-GAGTACA- GGTGTGCAGCTCTA-3’ and 5’-CAGCCCTACAGCCACATGAT-3’) as previously described^10^. Brains from corresponding fetuses were collected and the ventrolateral portions of caudal telencephalic gross sections containing the amygdala were microdissected under a dissecting microscope and stored in RNA*later* (Thermo Fisher Scientific, Waltham, MA). From each litter, two males and two females were selected by simple randomization for RNA isolation. Tissues were homogenized using stainless steel beads in a TissueLyser II apparatus (Qiagen, Hilden, Germany). For PN28 offspring, mice were sacrificed via cervical dislocations and brains were harvested, immediately frozen on dry ice, and stored at -80⁰C. Frozen brains were then submerged in RNA*later*-ICE (Thermo Fisher Scientific) and allowed to thaw overnight at -20⁰C per manufacturer’s protocol. Amygdalar dissections were performed as previously described^11^. Briefly, two coronal cuts were made at -1 mm and -2.75 mm with respect to bregma and the amygdala was microdissected from these slices under a dissecting microscope following delineations from the mouse brain atlas to ensure that there was no contamination from surrounding tissue. RNA from fetal brain and PN28 amygdalar dissections was purified using RNeasy Micro Kit (Qiagen) following manufacturer’s instructions.

**quantitative PCR (qPCR)**  RNA was converted to cDNA using the Quantitect Reverse Transcriptase Kit (Qiagen) according to manufacturer’s protocols and stored at -20⁰C. qPCR was performed using Taqman system with Taqman Gene Expression Master Mix (ThermoFisher Scientific) on each cDNA sample as previously described^1^ with minor modifications. Specific probes were used to quantify murine targets of interest (ThermoFisher Scientific) and GAPDH (Mm99999915_g1, ThermoFisher Scientific) was used as endogenous control. 12.5 ng of fetal or PN28 amygdala cDNA template were used per well and samples were run in duplicates. qPCR reactions were run on an Applied Biosystems StepOnePlus Real-Time PCR instrument (Applied Biosystems, Foster City, CA). Gene expression data were calculated using the ΔΔCt method.

**RNA-sequencing** RNA isolated from amygdalar microdissections from four control and four PS offspring (per sex) from different litters at PN28 was used for directional RNA-seq performed by the Genomics, Epigenomics, and Sequencing Core at the University of Cincinnati following previously published methods^12,13^. Briefly, RNA quality was determined by Bioanalyzer (Agilent, Santa Clara, CA). PolyA RNA was isolated using the NEBNext Poly(A) mRNA Magnetic Isolation Module (New England BioLabs, Ipswich, MA) with a total of 1 µg of good quality total RNA as input and enriched using the SMARTer Apollo NGS library prep system (Takara Bio USA, Mountain View, CA). Sequencing libraries were prepared using the NEBNext Ultr II Directional RNA Library Prep Kit for Illumina (New England Biolabs). After indexing, enrichment through 8 cycles of PCR, and passing initial quality control metrics, individually indexed and compatible libraries were proportionally pooled and sequenced using Nextseq 550 sequencer (Illumina, San Diego, CA). The sequencing setting of single read 1 × 85 bp to generate ∼50 M reads per sample was used. Sequencing data has been deposited in NCBI's Gene Expression Omnibus and is accessible through GEO Series accession number GSE189330.

### RNA-sequencing analysis RNA-seq reads were aligned to mouse genome, mm10, using STAR aligner^14^. Only uniquely aligned reads were retained for downstream analysis. Raw reads counts aligned to each genes were measured using FeatureCounts^15^. Differentially expressed genes (DEGs) were analyzed using RUVseq^16^ and EdgeR^17^. Specifically, we introduced two factors (k=1) of unwanted variation and estimated it using RUVs in RUVseq, which were incorporated into the model matrix for edgeR. Genes were defined as “expressed” if it displays > 0.5 CPM in at least one condition. Genes with fold-change > 1.5 and FDR < 0.05 were selected as differential genes for gene ontology analysis using EnrichR^18^. Top significant GO terms were selected for presentation.

**Statistics**  Data were analyzed by mixed linear factorial ANOVA with degrees of freedom calculated using the Kenward-Roger method (Proc Mixed, SAS version 9.4, SAS Institute, Cary, NC, USA), two-way ANOVA test followed by Tukey’s post hoc test (Prism 7.0c software; GraphPad Software, Inc., San Diego, CA, USA), or unpaired 2-tailed *t*-test (Prism 7.0c software) as indicated in figure legends. For behavioral and neuroendocrine assessments mixed linear ANOVA with PS x sex x CF model was used with litter as a randomized block factor. For fetal amygdala gene expression measurements, mixed linear ANOVA with PS x sex model and litter as a randomized block factor was used. qPCR validation of RNA seq data analysis was performed by Student’s *t*-test. Maternal behavior and milk CORT concentration analysis were performed by Student’s *t*-test. Effects of cross-fostering on amygdalar gene expression was analyzed by two-way ANOVA for PS and CF to determine main effects followed by Tukey’s post hoc test. P<0.05 was considered significant. The n represents either offspring, litter numbers, or dams, as indicated in figure legends. Results are reported as mean + standard error of the mean (s.e.m.).

**REFERENCES**

1. Zoubovsky SP, Hoseus S, Tumukuntala S, Schulkin JO, Williams MT, Vorhees CV, et al. Chronic psychosocial stress during pregnancy affects maternal behavior and neuroendocrine function and modulates hypothalamic CRH and nuclear steroid receptor expression. *Transl Psychiatry*. 2020; **10**: 6.

2. Amos-Kroohs RM, Williams MT, Braun AA, Graham DL, Webb CL, Birtles TS, et al. Neurobehavioral phenotype of C57BL/6J mice prenatally and neonatally exposed to cigarette smoke. *Neurotoxicol Teratol.* 2013; **35**: 34–45.

3. Kuerbitz J, Arnett M, Ehrman S, Williams MT, Vorhees CV, Fisher SE, et al. Loss of Intercalated Cells (ITCs) in the Mouse Amygdala of Tshz1 Mutants Correlates with Fear, Depression, and Social Interaction Phenotypes. *J Neurosci*. 2018; **38**:1160–1177.

4. Amos-Kroohs RM, Davenport LL, Gutierrez A, Hufgard JR, Vorhees CV, Williams MT. Developmental manganese exposure in combination with developmental stress and iron deficiency: Effects on behavior and monoamines. *Neurotoxicol Teratol*. 2016; **56**: 55–67.

5. Vorhees CV, Graham DL, Braun AA, Schaefer TL, Skelton MR, Richtand NM, et al. Prenatal immune challenge in rats: Effects of polyinosinic-polycytidylic acid on spatial learning, prepulse inhibition, conditioned fear, and responses to MK-801 and amphetamine. *Neurotoxicol Teratol*. 2015; **47**: 54–65.

6. Molet J, Heins K, Zhuo X, Mei YT, Regev L, Baram TZ., et al. Fragmentation and high entropy of neonatal experience predict adolescent emotional outcome. *Transl. Psychiatry.*2016; **6**: e702.

7. Davis EP, Stout SA, Molet J, Vegetabile B, Glynn LM, Sandman CA, et al. Exposure to unpredictable maternal sensory signals influences cognitive development across species. *Proc. Natl Acad. Sci.*2017; **39**: 10390–10395.

8. Baram TZ, Davis EP, Obenaus A, Sandman CA, Small SL, Solodkin A, et al. Fragmentation and unpredictability of early-life experience in mental disorders. *Am. J. Psychiatry.*2012; **9**: 907–915.

9. Chen Y, Wang J, Yang S, Utturkar S, Crodian J, Cummings S, et al. Effects of high-fat diet on secreted milk transcriptome in midlactation mice. *Physiol Genomics*. 2017; **49**: 757-762.

10. Mueller BR, Bale TL. Sex-specific programming of offspring emotionality after stress early in pregnancy. *J Neurosci*. 2008; **28**: 9055–9065.

11. Zapala MA, Hovatta I, Ellison JA, Wodicka L, Del Rio JA, Tennant R, et al. Adult mouse brain gene expression patterns bear an embryologic imprint. *Proc Natl Acad Sci U S A*. 2005; **102**: 10357–10362.

12. Rapp SJ, Dershem V, Zhang X, Schutte SC, Chariker ME. Varying Negative Pressure Wound Therapy Acute Effects on Human Split-Thickness Autografts. *J Burn Care Res*. 2020; **41**: 104–112.

13. Walsh KB, Zhang X, Zhu X, Wohleb E, Woo D, Lu L, et al. Intracerebral Hemorrhage Induces Inflammatory Gene Expression in Peripheral Blood: Global Transcriptional Profiling in Intracerebral Hemorrhage Patients. *DNA Cell Biol*. 2019; **38**: 660–669.

14. Dobin A, Davis CA, Schlesinger F, Drenkow J, Zaleski C, Jha S, et al. STAR: ultrafast universal RNA-seq aligner. *Bioinformatics*. 2013; **29**: 15–21.

15. Liao Y, Smyth GK, Shi W. featureCounts: an efficient general purpose program for assigning sequence reads to genomic features. *Bioinforma Oxf Engl*. 2014; **30**: 923–930.

16. Risso D. RUVSeq: Remove Unwanted Variation from RNA-Seq Data. 2014.

17. McCarthy DJ, Chen Y, Smyth GK. Differential expression analysis of multifactor RNA-Seq experiments with respect to biological variation. *Nucleic Acids Res*. 2012; **40**: 4288–4297.

18. Kuleshov MV, Jones MR, Rouillard AD, Fernandez NF, Duan Q, Wang Z, et al. Enrichr: a comprehensive gene set enrichment analysis web server 2016 update. *Nucleic Acids Res*. 2016; **44**: 90-97.

**Supplementary figure legends**

**Supplementary Table 1**. **Summary of maternal behavior analysis previously assessed in CTRL vs CGS dams.** Comparison of different quantitative and qualitative characteristics of maternal behavior previously assessed from postpartum day (PP) 2-6. CGS dams exhibit increased fragmentation of maternal care and increased unpredictability in maternal signals, as well as increased time to retrieve pups in pup retrieval task, when compared to CTRL dams.

**Supplementary Figure 1: Maternal behavior analysis in dams with CF offspring.** Percent time spent licking/grooming CF offspring (**a**), nursing CF offspring (**b**) and off nest (**c**) from postpartum day (PP) 2-5. Fragmentation in maternal care patterns quantified by mean duration (**d**) and number (**e**) of licking/grooming bouts recorded from PP2-PP5. Unpredictability in maternal care patterns quantified by entropy rates (**f**). Time to retrieve first CF pup (**g**) and all CF offspring (**h**) in pup retrieval task performed on PP6, CTRL dams with CF PS offspring = 9, CGS dams with CF CTRL offspring = 9. Data presented as mean + SEM. **p* < 0.05 unpaired 2-tailed *t*-test.

**Supplementary Figure 2. Psychosocial PS has no effect on offspring sociability or associative learning.** (**A**) Time spent exploring chamber in habituation phase and in testing phase with an unfamiliar conspecific during SI, CTRL offspring = 30, PS offspring = 29, PS offspring CF to CTRL mom = 39, CTRL offspring CF to CGS mom = 39. Freezing behavior measured during (**B**) training, (**C**) context and (**D**) cued phase of FC, CTRL offspring = 30, PS offspring = 29, PS offspring CF to CTRL mom = 39, CTRL offspring CF to CGS mom = 39. Data presented as mean + SEM. *****p* < 0.0001 mixed linear ANOVA with prenatal stress x cross-fostering x sex model and litter as a randomized block factor.

**Supplementary Figure 3: Maternal milk CORT concentrations in dams with CF offspring.** Maternal milk CORT measurements performed on PP9, CTRL dams with CF offspring = 9, CGS dams with CF offspring = 9. Data presented as mean + SEM. Unpaired 2-tailed *t*-test.

**Supplementary Figure 4. Effects of psychosocial PS on fetal brain development.** qPCR analysis of gene expression changes in molecular regulators of the HPA axis, including (**a**) CRH, (**b**) GR, (**c**) CRH R1, and (**d**) CRH R2, performed in E17.5 fetal amygdalas, N = 3 litters per group, 2 males and 2 females per litter. Data presented as mean + SEM. **p* < 0.05 mixed linear ANOVA with prenatal stress x sex model and litter as a randomized block factor.

**Supplementary Figure 5.** **Analysis of DEGs in the amygdala in CTRL females vs. CTRL males and PS males vs. PS females.** (**a**) Scatter plot displaying 9 female-specific and 9 male-specific genes in CTRL females vs. CTRL males comparison. (**b**) Summary of DEGs. Genes were considered significant with an FDR < 0.05 and FC > 1.5. (**c**) Significantly enriched pathways after gene ontology analysis of male-specific genes using molecular function category. Genes significantly expressed in the amygdala of CTRL males when compared to CTRL females include genes encoding for calcium binding proteins associated with RAGE receptor binding process and long chain fatty acid binding process. (**d**) Scatter plot displaying 344 PS female-specific and 173 PS male-specific genes. Genes were considered significant with an FDR < 0.05 and FC > 1.5. (**e**) Significantly enriched pathways after gene ontology analysis of PS female-specific genes using biological process and cellular component categories.

**Supplementary Table 2**. **Comparison of fold change determined by RNA-seq and qPCR for a subset of genes selected for validation of RNA-seq and summary of CF-induced changes.** Comparison of fold changes by RNA-seq and qPCR shows a positive correlation. Summary of gene expression changes obtained after CF obtained via qPCR has also been provided.
