# Supplementary material for: Neurobehavioral abnormalities following prenatal psychosocial stress are differentially modulated by maternal environment
Source: Transl Psychiatry. 2022 Jan 17;12:22. doi: 10.1038/s41398-022-01785-5 (PMC8764031; doi:10.1038/s41398-022-01785-5)
Supplement: Supplementary file 2 — Supplementary table 1 [file 41398_2022_1785_MOESM2_ESM.pptx]

## Slide 1
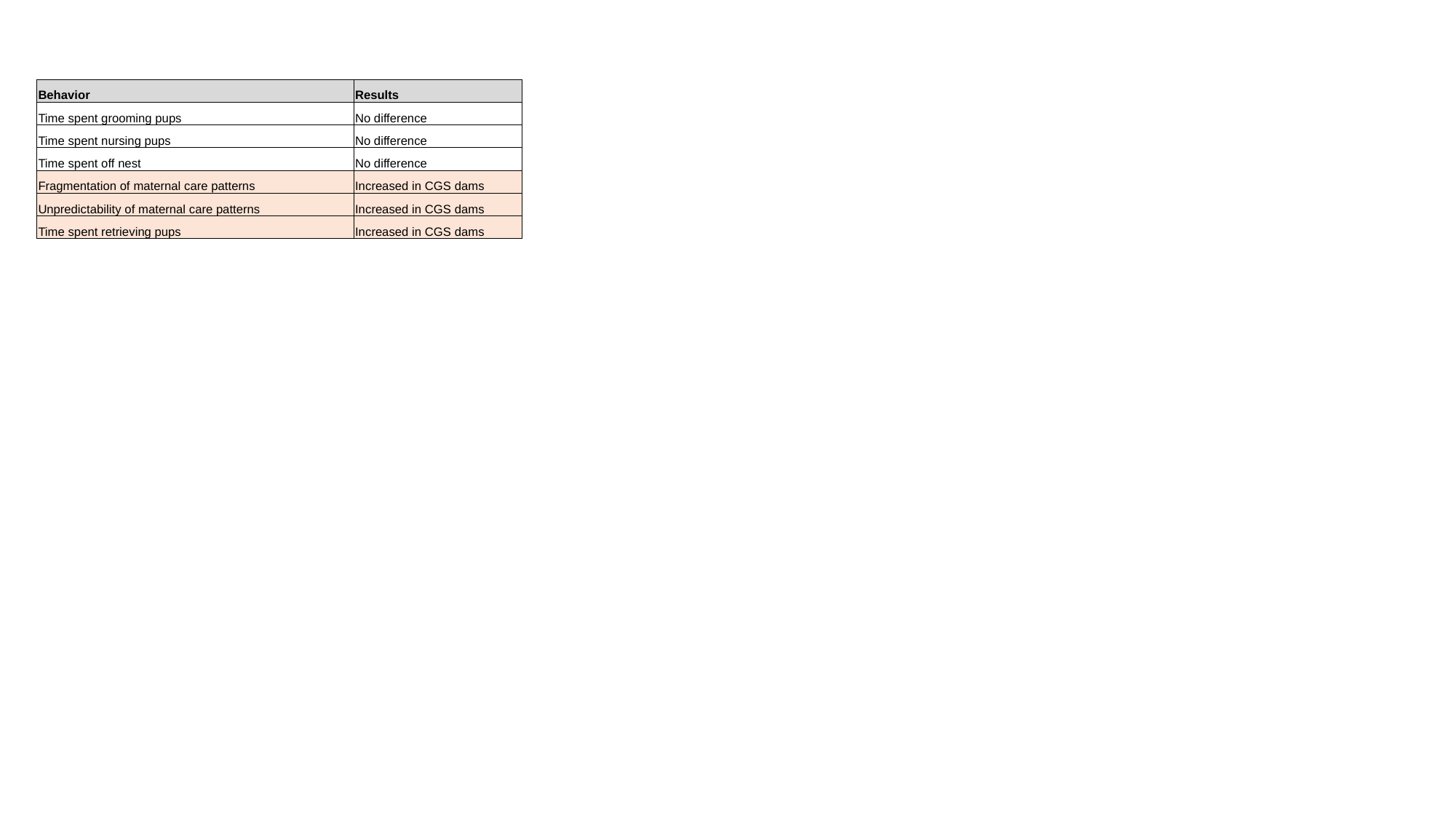

| Behavior | Results |
| --- | --- |
| Time spent grooming pups | No difference |
| Time spent nursing pups | No difference |
| Time spent off nest | No difference |
| Fragmentation of maternal care patterns | Increased in CGS dams |
| Unpredictability of maternal care patterns | Increased in CGS dams |
| Time spent retrieving pups | Increased in CGS dams |
