# Supplementary material for: Neurobehavioral abnormalities following prenatal psychosocial stress are differentially modulated by maternal environment
Source: Transl Psychiatry. 2022 Jan 17;12:22. doi: 10.1038/s41398-022-01785-5 (PMC8764031; doi:10.1038/s41398-022-01785-5)
Supplement: Supplementary file 7 — Supplementary figure 5 [file 41398_2022_1785_MOESM7_ESM.pptx]

## Slide 1
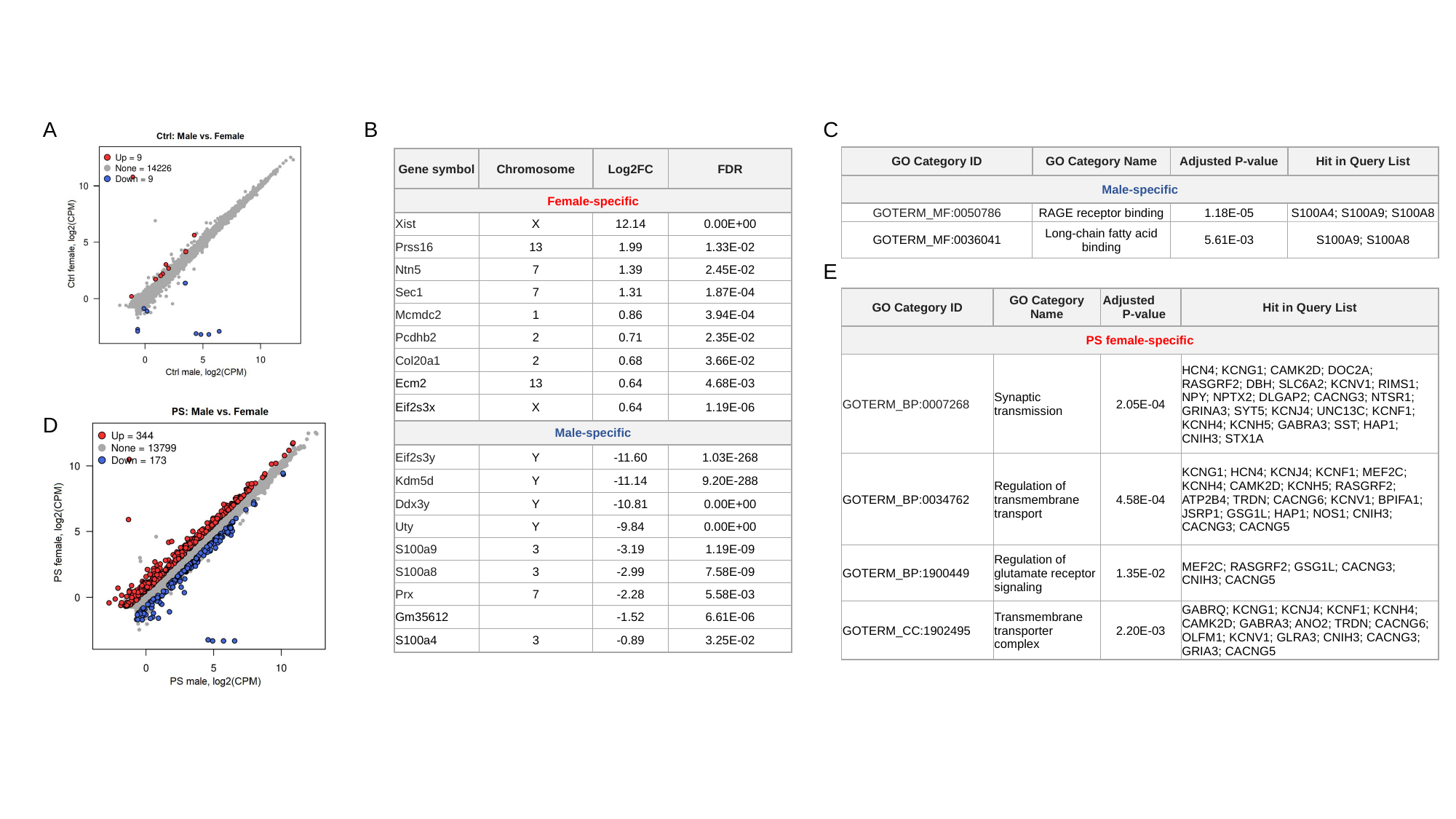

A
B
C
| GO Category ID | GO Category Name | Adjusted P-value | Hit in Query List |
| --- | --- | --- | --- |
| Male-specific | | | |
| GOTERM\_MF:0050786 | RAGE receptor binding | 1.18E-05 | S100A4; S100A9; S100A8 |
| GOTERM\_MF:0036041 | Long-chain fatty acid binding | 5.61E-03 | S100A9; S100A8 |
| Gene symbol | Chromosome | Log2FC | FDR |
| --- | --- | --- | --- |
| Female-specific | | | |
| Xist | X | 12.14 | 0.00E+00 |
| Prss16 | 13 | 1.99 | 1.33E-02 |
| Ntn5 | 7 | 1.39 | 2.45E-02 |
| Sec1 | 7 | 1.31 | 1.87E-04 |
| Mcmdc2 | 1 | 0.86 | 3.94E-04 |
| Pcdhb2 | 2 | 0.71 | 2.35E-02 |
| Col20a1 | 2 | 0.68 | 3.66E-02 |
| Ecm2 | 13 | 0.64 | 4.68E-03 |
| Eif2s3x | X | 0.64 | 1.19E-06 |
| Male-specific | | | |
| Eif2s3y | Y | -11.60 | 1.03E-268 |
| Kdm5d | Y | -11.14 | 9.20E-288 |
| Ddx3y | Y | -10.81 | 0.00E+00 |
| Uty | Y | -9.84 | 0.00E+00 |
| S100a9 | 3 | -3.19 | 1.19E-09 |
| S100a8 | 3 | -2.99 | 7.58E-09 |
| Prx | 7 | -2.28 | 5.58E-03 |
| Gm35612 | | -1.52 | 6.61E-06 |
| S100a4 | 3 | -0.89 | 3.25E-02 |
E
| | | | |
| --- | --- | --- | --- |
| GO Category ID | GO Category Name | Adjusted P-value | Hit in Query List |
| PS female-specific | | | |
| GOTERM\_BP:0007268 | Synaptic transmission | 2.05E-04 | HCN4; KCNG1; CAMK2D; DOC2A; RASGRF2; DBH; SLC6A2; KCNV1; RIMS1; NPY; NPTX2; DLGAP2; CACNG3; NTSR1; GRINA3; SYT5; KCNJ4; UNC13C; KCNF1; KCNH4; KCNH5; GABRA3; SST; HAP1; CNIH3; STX1A |
| GOTERM\_BP:0034762 | Regulation of transmembrane transport | 4.58E-04 | KCNG1; HCN4; KCNJ4; KCNF1; MEF2C; KCNH4; CAMK2D; KCNH5; RASGRF2; ATP2B4; TRDN; CACNG6; KCNV1; BPIFA1; JSRP1; GSG1L; HAP1; NOS1; CNIH3; CACNG3; CACNG5 |
| GOTERM\_BP:1900449 | Regulation of glutamate receptor signaling | 1.35E-02 | MEF2C; RASGRF2; GSG1L; CACNG3; CNIH3; CACNG5 |
| GOTERM\_CC:1902495 | Transmembrane transporter complex | 2.20E-03 | GABRQ; KCNG1; KCNJ4; KCNF1; KCNH4; CAMK2D; GABRA3; ANO2; TRDN; CACNG6; OLFM1; KCNV1; GLRA3; CNIH3; CACNG3; GRIA3; CACNG5 |
D
