# Supplementary material for: Neurobehavioral abnormalities following prenatal psychosocial stress are differentially modulated by maternal environment
Source: Transl Psychiatry. 2022 Jan 17;12:22. doi: 10.1038/s41398-022-01785-5 (PMC8764031; doi:10.1038/s41398-022-01785-5)
Supplement: Supplementary file 8 — Supplementary table 2 [file 41398_2022_1785_MOESM8_ESM.pptx]

## Slide 1
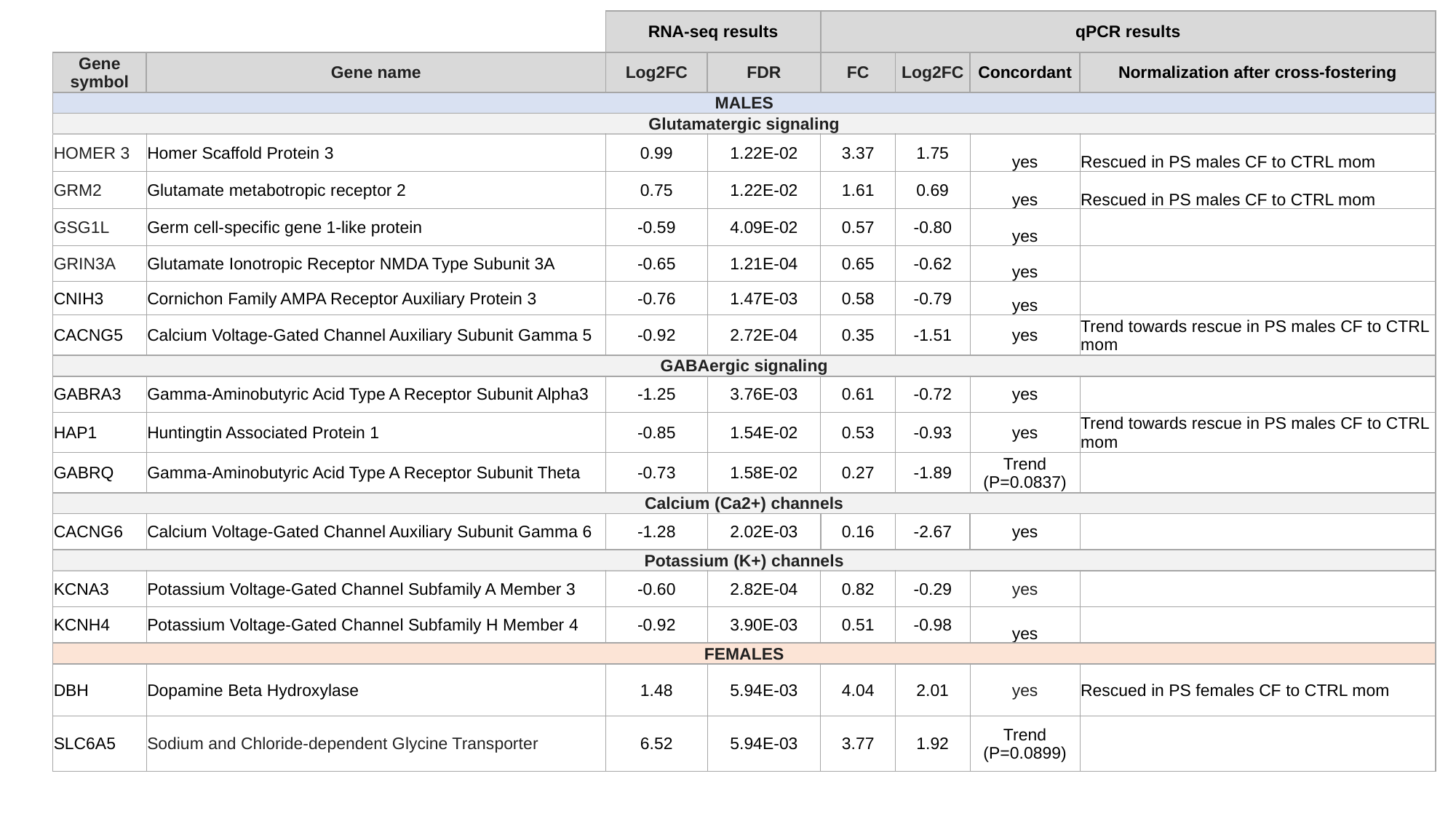

| | | RNA-seq results | | qPCR results | | | |
| --- | --- | --- | --- | --- | --- | --- | --- |
| Gene symbol | Gene name | Log2FC | FDR | FC | Log2FC | Concordant | Normalization after cross-fostering |
| MALES | | | | | | | |
| Glutamatergic signaling | | | | | | | |
| HOMER 3 | Homer Scaffold Protein 3 | 0.99 | 1.22E-02 | 3.37 | 1.75 | yes | Rescued in PS males CF to CTRL mom |
| GRM2 | Glutamate metabotropic receptor 2 | 0.75 | 1.22E-02 | 1.61 | 0.69 | yes | Rescued in PS males CF to CTRL mom |
| GSG1L | Germ cell-specific gene 1-like protein | -0.59 | 4.09E-02 | 0.57 | -0.80 | yes | |
| GRIN3A | Glutamate Ionotropic Receptor NMDA Type Subunit 3A | -0.65 | 1.21E-04 | 0.65 | -0.62 | yes | |
| CNIH3 | Cornichon Family AMPA Receptor Auxiliary Protein 3 | -0.76 | 1.47E-03 | 0.58 | -0.79 | yes | |
| CACNG5 | Calcium Voltage-Gated Channel Auxiliary Subunit Gamma 5 | -0.92 | 2.72E-04 | 0.35 | -1.51 | yes | Trend towards rescue in PS males CF to CTRL mom |
| GABAergic signaling | | | | | | | |
| GABRA3 | Gamma-Aminobutyric Acid Type A Receptor Subunit Alpha3 | -1.25 | 3.76E-03 | 0.61 | -0.72 | yes | |
| HAP1 | Huntingtin Associated Protein 1 | -0.85 | 1.54E-02 | 0.53 | -0.93 | yes | Trend towards rescue in PS males CF to CTRL mom |
| GABRQ | Gamma-Aminobutyric Acid Type A Receptor Subunit Theta | -0.73 | 1.58E-02 | 0.27 | -1.89 | Trend (P=0.0837) | |
| Calcium (Ca2+) channels | | | | | | | |
| CACNG6 | Calcium Voltage-Gated Channel Auxiliary Subunit Gamma 6 | -1.28 | 2.02E-03 | 0.16 | -2.67 | yes | |
| Potassium (K+) channels | | | | | | | |
| KCNA3 | Potassium Voltage-Gated Channel Subfamily A Member 3 | -0.60 | 2.82E-04 | 0.82 | -0.29 | yes | |
| KCNH4 | Potassium Voltage-Gated Channel Subfamily H Member 4 | -0.92 | 3.90E-03 | 0.51 | -0.98 | yes | |
| FEMALES | | | | | | | |
| DBH | Dopamine Beta Hydroxylase | 1.48 | 5.94E-03 | 4.04 | 2.01 | yes | Rescued in PS females CF to CTRL mom |
| SLC6A5 | Sodium and Chloride-dependent Glycine Transporter | 6.52 | 5.94E-03 | 3.77 | 1.92 | Trend (P=0.0899) | |
